# Evaluation of Apoptotic Gene Expression in Hepatoma Cell Line (HepG2) Following Nisin Treatment

**DOI:** 10.31557/APJCP.2021.22.5.1413

**Published:** 2021-05

**Authors:** Nahid Zainodini, Mohammad Reza Hajizadeh, Mohammad Reza Mirzaei

**Affiliations:** 1 *Immunology of Infectious Diseases Research Center, Research Institute of Basic Medical Sciences, Rafsanjan University of Medical Sciences, Rafsanjan, Iran. *; 2 *Molecular Medicine Research Center, Research Institute of Basic Medical Sciences, Rafsanjan University of Medical Sciences, Rafsanjan, Iran. *; 3 *Department of Clinical Biochemistry, Faculty of Medicine, Rafsanjan University of Medical Sciences, Rafsanjan, Iran. *

**Keywords:** Liver cancer, antimicrobial peptide, programmed cell death, cancer treatmen, nisin

## Abstract

**Objective:**

The present study aims to examine the effects of nisin on the survival and apoptosis of the hepatoma cell line HepG2 and to investigate possible apoptosis pathways activated by nisin.

**Materials and Methods:**

For this purpose, viability and apoptosis of the cells were accomplished by the nisin treatment using the MTT assay and Annexin-V-fluorescein/propidium iodide (PI) double staining, respectively. Additionally, the human apoptosis PCR array was performed to determine pathways or genes activated by nisin during possible apoptosis.

**Results:**

The results of the present study showed that nisin was able to decrease cell viability (IC_50_ ~ 40 µg/ml) in a dose-dependent manner and could induce apoptosis in HepG2 cells. PCR data indicated a considerable increase in the expression of genes, such as caspase and BCL2 families, involved in the induction of apoptosis.

**Conclusions:**

The data from this study showed that overexpression of genes involved in the intrinsic pathway of apoptosis, especially caspase-9 and BID, increased apoptosis in HepG2 cells treated by nisin, compared to the control group.

## Introduction

Hepatocellular carcinoma is the third-most prevalent cause of cancer-related deaths leading to approximately half a million annual deaths across the world. Besides, it is referred to as the primary malignant neoplasm of epithelial liver cells. The prevalence of hepatocellular carcinoma is increasing in the western countries. However, this disease has been a serious health problem in Asia and Africa for a long time (Lin et al., 2017; Ayuso et al., 2018; Taskaeva and Bgatova, 2018).

In the current clinical practice, the main risk factors of hepatocellular carcinoma were primarily associated with suppressed hepatitis B virus during the treatment, sustained virologic response after hepatitis C, as well as alcoholic and non-alcoholic fatty liver disease (Kulik and El-Serag, 2019). Chemotherapy and surgical procedures are the most prevalent clinical treatments for hepatocellular carcinoma. Thus, novel and efficient medicines are required to be discovered for human hepatoma (Chen et al., 2015; Ayuso et al., 2018). 

Peptides with antimicrobial activity are archaic evolutionary weapons. Besides, their broad distribution across the species of animals and plants indicates that antimicrobial peptides play a key role in the effective development of complex multicellular organisms (Zasloff, 2002). Nisin is an antimicrobial peptide composed of particular gram-positive bacteria, such as the species Streptococcus and Lactococcus (Lubelski et al., 2009). It was originally identified in milk fermentation culture in 1928 and was commercialized as an antimicrobial component in England in 1953 (Delves-Broughton et al., 1996). The safe usage of nisin was approved in 1969 as bacterial blockage in human foods (Shin et al., 2016).

Antimicrobial peptides have been studied for their therapeutic effects, such as their ability to perform several biological tasks, including antiviral properties, DNA synthesis, inhibition of membrane protein synthesis, and apoptosis or cytotoxicity of tumor cells (Cornut et al., 2008; Hamedeyazdan et al., 2012). To put it differently, antimicrobial peptides have been examined as potential therapeutic medicines for such features (Yusuf et al., 2014). This study concentrates on examining effects of nisin on the HepG2 apoptosis pathway as a cell line of hepatocellular carcinoma.

## Materials and Methods

*Cell culture*

The National Cell Bank of the Pasteur Institute (Tehran, Iran) provided the human liver cancer cell line (HepG2). Following incubation in a humidified atmosphere with 5% CO_2_ and 95% air at 37°C, RPMI 1640 (Gibco) with 10% fetal bovine serum (FBS) (Gibco), streptomycin (100 μg/ml), as well as penicillin (100 U/ml) were applied to the cell culture.

*Nisin preparation*

Four mg of nisin (Sigma-Aldrich) was dissolved in 1ml of HCl 0.02 N to get a concentration of 4mg/ml for the final preparation of nisin. The major concentration was sterilized, and 500μl of the major sample concentration was added into the first tube and blended with 500μl of the RPMI 1640 medium. Next, through serial dilution for obtaining other concentrations, 200µl of the volume was moved from the first 2ml tubes into the last tube.

*MTT assay*

Assay 3-(4, 5-dimethylthiazol-2-yl)-2, 5-diphenyltetrazoliumbromide (MTT) (Sigma-Aldrich) was used to assess cell viability. In 96-well plates (3 × 103 cells per well in the 200μl RPMI 1640 medium), HepG2 cells were seeded and incubated at 37°C overnight. Later on, the cells were treated with 200μl of the fresh medium at various nisin concentrations (1-200µg/ml) for 24 and 48 h. Next, a total of 20μl of the MTT solution was added to every well, and the plates were incubated after the treatment. After the 4h incubation, the culture medium was detached to dissolve formazan crystals, with 100μl of DMSO added to every well. Next, the sample’s absorption was read at 570nm with a 630nm reference filter. The process was repeated three times. Besides, (Sample/control) × 100 was performed to define cell viability. 

*Apoptosis analysis*

The Annexin-V-FLUOS staining kit (Roche Diagnostics) was employed to assess apoptosis induction by nisin, in accordance with the manufacturer’s guidelines. This double staining technique was used to separate apoptotic cells from necrotic ones. In brief, the cells were treated with two different nisin concentrations (10 and 25 µg/ml) at 37°C for 24h. With PBS, 1 × 10^6^ cells were washed and centrifuged at 200g for 5min after the treatment. The cells were suspended in 100µl of Annexin-V-FLUOS, and the solution was tagged with the Annexin-V-FLUOS reagent and propidium iodide. The cell suspensions were incubated for 15 min at 25°C. Next, triplicate specimens were examined on a BD FACS Calibur flow cytometer.

*RT2 Profiler PCR array*

To investigate a gene expression panel associated with the apoptosis path (Human Apoptosis PCR Array, Catalog No. PAHS-012), the RT2 Profiler TM PCR array (SABiosciences) was employed. Besides, 84 apoptosis-related genes, housekeeping genes, as well as RNA and PCR quality controls were included in the array. The cells were either treated with a medium of nisin 0.25µg/ml or the control at 37°C for 24h. Besides, the cells were washed twice with the PBS and then disrupted in the 500μl RLT buffer. Next, the cell lysis was transferred to the RNeasy Mini spin column after adding ethanol. In the end, the membrane-bound RNAs were washed by centrifuging the columns using the RNeasy Mini kit (Qiagen). To evaluate RNA purity and integrity, nanodrop and agarose gel electrophoresis were employed, respectively. Besides, the RT2 First Strand Kit (Qiagen) from 1.0µg of the total RNA for the 96-well plate was employed to generate the complementary DNA (cDNA). Next, the mixture of the GE Buffer and RNA samples was incubated at 37°C (5 min) to eliminate genomic DNA. The samples were then reverse-transcribed using BC4 Reverse Transcriptase and the Bio-rad C1000 thermal cycler, under conditions of 42°C (15 min), 95°C (5 min), and 4°C holding. For the real-time PCR analysis, a CFX96 Real-Time PCR Detection System (Bio-Rad, USA) and the following conditions were employed. The first cycle was done at 95°C for 10 min, and 40 cycles at 95 °C for 15s, 60°C for 40s, and 72°C for 30s.

*Statistical analysis*

All tests were performed three times. Besides, the data on cell survival and apoptosis were analyzed by the one-way analysis of variance (ANOVA) using SPSS 16.0 (SPSS, Chicago, IL, USA). The mean ± standard deviation (SD) were measured, and the difference appeared to be statistically significant at P ˂ 0.05. In addition, the web-based software of RT2 Profiler PCR Array version 3.5 (http://pcrdataanalysis.sabiosciences.com/pcr/arrayanalysis.php) was employed to export and analyze the values of the resulting cycle threshold (CT) for gene expression. Besides, the ∆∆CT technique was employed to calculate fold changes.

## Results

*Nisin effects on cell viability*

**Figure 1:**
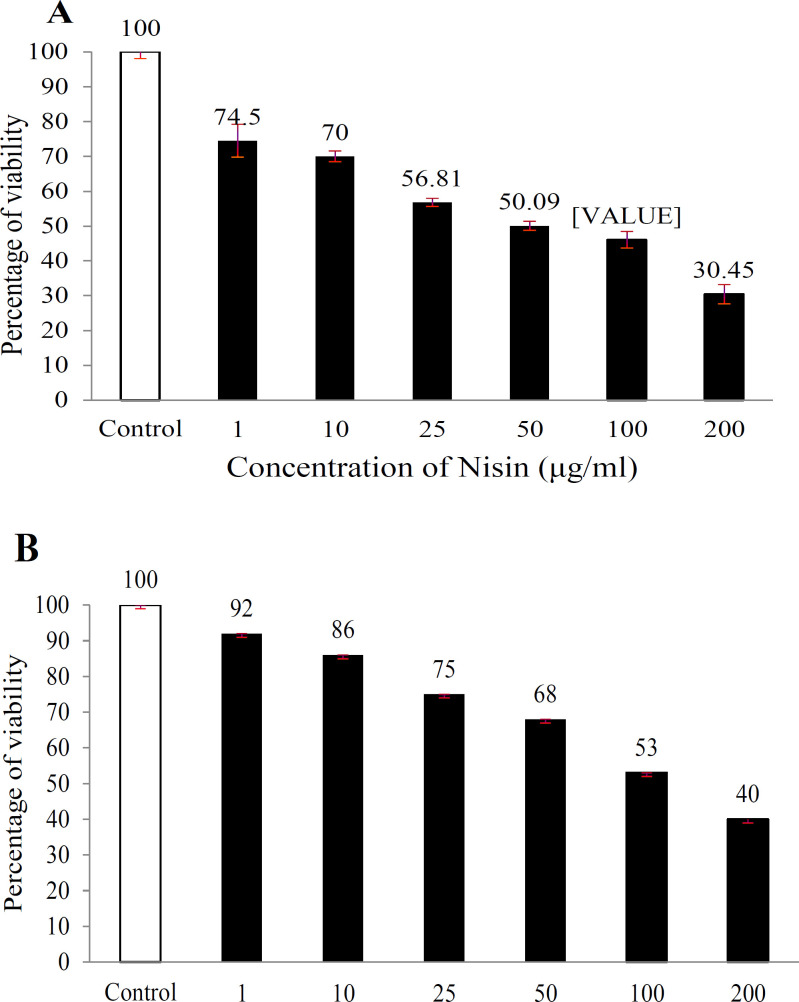
The Effect of Nisin on of HepG2 Cell's Survival. Various concentrations of nisin was applied for 24 (A), 48 (B) and then MTT assay was done for the measurement of the cell viability (%). The average of each tripliate experiment is presented in individual column as mean ± SD

To assess potential cytotoxicity of nisin in contact with the HepG2 cell line, the MTT viability assay was employed. Accordingly, various concentrations of nisin (1, 10, 25, 50, 100, and 200 µg/ml) were employed in the present research. The viability rate of the treated cells displayed a reduction following an increase in the dose as against the control group after 24 and 48h of incubation (Figure 1). The results revealed that viability declined significantly (P ˂ 0.05) from 1 µg/ml to 200 µg/ml with an IC_50_ value of around 40 µg/ml. In contrast, no significant difference was observed between the concentration of 1µg/ml and that of the control group. Thus, nisin could be used as a growth inhibitor for HepG2 cells. 

*Nisin effects on apoptosis*

**Figure 2:**
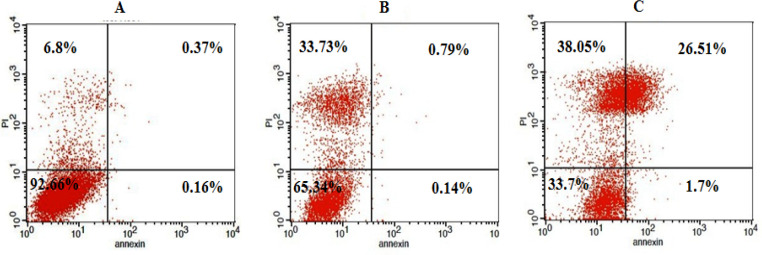
Flow Cytometric Analysis of HepG2 Cells after 24 h Treatment with Medium (A), 10 µg/ml nisin (B), and 25 µg/ml nisin (C). Scatter plots consist of four quadrants: upper left (Annexin-V-/PI+, necrotic cells), upper right (Annexin-V+/PI+, late apoptotic cells), lower left (Annexin-V-/PI-, viable cells), lower right (Annexin-V+/PI-, early apoptotic cells).

Annexin-V-fluorescein/propidium iodide (PI) double staining was performed to examine apoptosis induction by nisin in HepG2 cells. The quantity of apoptotic cells increased as against the untreated group after being subjected to 25µg/ml of nisin for 24h. In the untreated control tube, the population of viable cells was 92.66%, following adding 25µg/ml of nisin, and about a quarter of the cells underwent late apoptosis (Figure 2). 

*Nisin effects on apoptosis-related genes*

Real-time PCR was performed using the Human Apoptosis RT² Profiler PCR Array to express main genes engaged in programmed cell death, which responded to the 24h treatment (25µg/ml) of nisin. Besides, several members of the families, including the domain proteins of *TNF/TNFR, BCL2/BAG, BIR, TRAF*, as well as caspases were examined. In addition, genes with fold-change values of more than 2 and less than 0.5 were expressed in different manners. Table 1 shows fold changes of the genes studied on the PCR array. A total of 13 out of all chosen genes, including *BAD, BAK1, BCLAF1, BNIP3L, CARD6, CASP5, CASP6, CASP10, CFLAR, FADD, HRK, LTBR*, as well as TNF were upregulated. In contrast, 17 genes, including *NOD1, TP53, TP73, NAIP, BIRC3, BCL2A1, BAG1, BAG3, BAG, TNFRSF25, CRADD, TNFRSF10B, CASP3, BRAF, BIK, APAF1*, and *BCL2* were downregulated. 

**Table 1:** Assessment of Apoptosis-Related Genes which are Expressed in HepG2 Cells Following Nisin Treatment for 24 h

Gene symbol	Protein/ gene name	Activity	Fold change
*APAF1*	Apoptotic Peptidase Activating Factor 1	Pro-apoptosis	-2.51
*BAD*	BCL2-associated agonist of cell death	Pro-apoptosis	1.45
*BID*	BH3 interacting domain death agonist	Pro-apoptosis	2.66
*BIK*	BCL2 Interacting Killer	Pro-apoptosis	-4.89
*BCL10*	B-Cell CLL/Lymphoma 10	Pro-apoptosis	-1.58
*BCL2L11*	BCL2 Like 11	Pro-apoptosis	-1.22
*BCLAF1*	BCL2 Associated Transcription Factor 1	Pro-apoptosis	2.23
*BNIP2*	BCL2 Interacting Protein 2	Pro-apoptosis	1.17
*BNIP3*	BCL2 Interacting Protein 3	Pro-apoptosis	-1.13
*BNIP3L*	BCL2 Interacting Protein 3 Like	Pro-apoptosis	2.34
*BRAF*	B-Raf Proto-Oncogene, Serine/Threonine Kinase	Pro-apoptosis	-4.26
*CARD6*	Caspase Recruitment Domain Family Member 6	Pro-apoptosis	3.27
*CARD8*	Caspase Recruitment Domain Family Member 8	Pro-apoptosis	1.79
*CASP1*	Caspase 1, Apoptosis-Related Cysteine Peptidase	Pro-apoptosis	10.94
*CASP2*	Caspase 2, Apoptosis-Related Cysteine Peptidase	Pro-apoptosis	-1.51
*CASP3*	Caspase 3, Apoptosis-Related Cysteine Peptidase	Pro-apoptosis	-2.03
*CASP4*	Caspase 4, Apoptosis-Related Cysteine Peptidase	Pro-apoptosis	1.24
*CASP5*	Caspase 5, Apoptosis-Related Cysteine Peptidase	Pro-apoptosis	2.29
*CASP6*	Caspase 6, Apoptosis-related cysteine peptidase	Pro-apoptosis	9.72
*CASP7*	Caspase 7, Apoptosis-related cysteine peptidase	Pro-apoptosis	28.83
*CASP8*	Caspase 8, Apoptosis-related cysteine peptidase	Pro-apoptosis	-1.46
*CASP9*	Caspase 9, Apoptosis-related cysteine peptidase	Pro-apoptosis	70.7
*CASP10*	Caspase 10, Apoptosis-related cysteine peptidase	Pro-apoptosis	5.11
*CASP14*	Caspase 14, Apoptosis-related cysteine peptidase	Pro-apoptosis	1.56
*CD27*	CD27 Molecule	Pro-apoptosis	1.12
*CD40*	CD40 Molecule	Pro-apoptosis	-1.3
*CD40LG*	CD40 Ligand	Pro-apoptosis	1.22
*CD70*	CD70 Molecule	Pro-apoptosis	1.62
*CFLAR*	CASP8 and FADD-like apoptosis regulator	Pro-apoptosis	-2.99
*CIDEA*	Cell Death Inducing DFFA Like Effector A	Pro-apoptosis	-1.28
*CIDEB*	Cell Death Inducing DFFA Like Effector B	Pro-apoptosis	-1.36
*CRADD*	CASP2 And RIPK1 Domain Containing Adaptor With Death Domain	Pro-apoptosis	-3.61
*DAPK1*	Death Associated Protein Kinase 1	Pro-apoptosis	-10.38
*DFFA*	DNA fragmentation factor, 45kDa, alpha polypeptide	Pro-apoptosis	1.19
*FADD*	Fas Associated Via Death Domain	Pro-apoptosis	2.49
*FAS*	Fas (TNF receptor superfamily, member 6)	Pro-apoptosis	-1.14
*FASLG*	Fas ligand (TNF superfamily, member 6)	Pro-apoptosis	-1.14
*HRK*	Harakiri, BCL2 Interacting Protein	Pro-apoptosis	3.42
*LTA*	Lymphotoxin Alpha	Pro-apoptosis	-1.53
*LTBR*	Lymphotoxin Beta Receptor	Pro-apoptosis	3.26
*NOD1*	Nucleotide Binding Oligomerization Domain Containing 1	Pro-apoptosis	-2.25
*PYCARD*	PYD And CARD Domain Containing	Pro-apoptosis	-1.64
*TNF*	Tumor Necrosis Factor	Pro-apoptosis	6.62
*TNFSF8*	Tumor necrosis factor (ligand) superfamily, member 8	Pro-apoptosis	-1.14
*TNFSF10*	Tumor necrosis factor (ligand) superfamily, member 10	Pro-apoptosis	1.14
*TNFRSF1A*	Tumor necrosis factor receptor superfamily, member 1A	Pro-apoptosis	10.27
*TNFRSF10A*	TNF Receptor Superfamily Member 10a	Pro-apoptosis	-1.14
*TNFRSF10B*	TNF Receptor Superfamily Member 10b	Pro-apoptosis	-2.53
*Gene symbol*	Protein/ gene name	Activity	Fold change
*TNFRSF9*	Tumor necrosis factor receptor superfamily, member 9	Pro-apoptosis	1.55
*TNFRSF11B*	Tumor necrosis factor receptor superfamily, member 11b	Pro-apoptosis	-1.14
*TNFRSF21*	Tumor necrosis factor receptor superfamily, member 21	Pro-apoptosis	-1.14
*TNFRSF25*	TNF Receptor Superfamily Member 25	Pro-apoptosis	-6.612
*TRADD*	TNFRSF1A-associated via death domain	Pro-apoptosis	-1.14
*BAK1*	BCL2-antagonist/killer 1	Anti-apoptosis	2.47
*BAX*	BCL2 Associated X, Apoptosis Regulator	Anti-apoptosis	-1.83
*BAG1*	BCL2 Associated Athanogene 1	Anti-apoptosis	2.99
*BAG3*	BCL2 Associated Athanogene 3	Anti-apoptosis	3.28
*BAG4*	BCL2-associated Athanogene 4	Anti-apoptosis	2.36
*BCL2*	B-cell CLL/lymphoma 2	Anti-apoptosis	-2.59
*BCL2A1*	BCL2 Related Protein A1	Anti-apoptosis	5.76
*BCL2L2*	BCL2 Like 2	Anti-apoptosis	1.48
*BCL2L10*	BCL2-like 10 (apoptosis facilitator)	Anti-apoptosis	-2.8
*BFAR*	Bifunctional Apoptosis Regulator	Anti-apoptotic	-1.09
*BIRC3*	Baculoviral IAP repeat containing 3	Anti-apoptosis	-3.56
*BIRC6*	Baculoviral IAP repeat containing 6	Anti-apoptosis	-1.97
*BIRC8*	Baculoviral IAP repeat containing 8	Anti-apoptosis	-1.21
*BNIP1*	BCL2 Interacting Protein 1	Anti-apoptosis	1.11
*IGF1R*	Insulin Like Growth Factor 1 Receptor	Anti-apoptosis	-1.17
*MCL1*	Myeloid Cell Leukemia Sequence 1 (BCL2-Related)	Anti-apoptosis	1.95
*NAIP*	NLR Family Apoptosis Inhibitory Protein	Anti-apoptosis	-2.6
*NOL3*	Nucleolar protein 3 (apoptosis repressor with CARD domain)	Anti- apoptosis	-1.57
*RIPK2*	Receptor-interacting serine-threonine kinase 2	Anti-apoptosis	-1.23
*TP53*	Tumor protein p53	Anti-apoptosis	-1.74
*TP73*	Tumor protein p73	Anti-apoptosis	-4.15
*TRAF2*	TNF receptor-associated factor 2	Anti-apoptosis	-2.21
*TRAF3*	TNF receptor-associated factor 3	Anti-apoptosis	-2.67
*TRAF4*	TNF receptor-associated factor 4	Anti-apoptosis	-3.52
*XIAP*	X-Linked Inhibitor of Apoptosis	Anti-apoptosis	1.3

## Discussion

Given the low prognosis of liver cancer and inefficiency of current treatments for all types of this disorder, development of new anticancer agents for this cancer is of great importance (Anwanwan et al., 2020). Cancer cells show unlimited growth behavior; in other words, they strongly suppress apoptosis (programmed cell death). Moreover, deficiencies in the apoptotic pathway regulation, in addition to cancer development, may lead to resistance to cancer chemotherapy. One of the cancer-fighting approaches is the development of novel therapeutic agents that either upregulate pro-apoptotic molecules or downregulate anti-apoptotic molecules (Fesik, 2005; Baskić et al., 2006; Call et al., 2008). In the late 1970s, research reported anti-cancerous characteristics for bacteriocins. They are, therefore, known to be favorable alternatives for developing anticancer composites (Breukink and de Kruijff, 1999; Bishayee and Sethi, 2016). Accordingly, potential apoptotic effects of nisin against HepG2 cells were investigated in this research. The PCR array technology was employed to define molecular mechanisms of nisin-induced apoptosis. According to the findings, nisin decreased cell viability and induced apoptosis. There is a small number of research conducted on the anti-tumoral activity of nisin (Lagos et al., 2009; Shaikh et al., 2012; Yates et al., 2012). Joo et al., (2012) investigated the role of nisin in inducing apoptosis of head and neck squamous cell carcinoma (HNSCC). Their findings revealed that nisin could increase apoptosis by the upregulation of CHAC1, i.e. a regulatory protein responsible for cation transport and apoptosis mediation, in HNSCC cells receiving nisin, as opposed to the control group. Another study in 2018 examined the effects of nisin on the genes involved in the metastasis pathway on colon cancer cell lines. The results showed that the expression of CEA and MMPs (as main genes in the start point of metastasis) underwent a reduction after nisin treatment of colon cancer cell lines (Norouzi et al., 2018). 

Based on the study of lewies et al., (2018) human malignant melanoma cells experienced toxic conditions after receiving nisin Z, compared to non-malignant keratinocytes. Besides, nisin Z reduced proliferation and invasion of cancer cells by increasing apoptosis and producing reactive oxygen species.

Following the use of the qPCR array technology, it was found out that nisin could be capable of changing the expression of apoptosis-related genes. This PCR array consisted of a number of apoptosis-involved gene families, such as caspase, *TRAF, Bcl-2*, and *IAP*, as well as* TNF* ligands and their receptors. In this study, the data suggested different gene expressions in the signaling pathways of extrinsic and intrinsic apoptosis. 

The present study demonstrated that nisin is capable of regulating and modifying the apoptosis pathway (intrinsic and extrinsic). Accordingly, nisin could affect this pathway, particularly by upregulation of pro-apoptosis genes. As a result, a total of 14 genes of the pro-apoptotic genes were increased, which included *BID, BCLAF1, BNIP3L, CARD6*, caspase family members (1, 5, 6, 7, 10), *FADD, HRK, LTBR, TNF, and TNFRSF1A*. In contrast, 9 of other genes, including *APAF1, BIK, BRAF, CASP3, CFLAR, CRADD, NOD1, TNFRSF10B*, and *TNFRSF2* underwent downregulation. Similarly, anti-apoptotic genes were affected by nisin, with most of which having shown decreased expression. Accordingly, *BCL2, BCL2L10, BIRC3, NAIP, TP73, TRAF2, TRAF3*, and *TRAF4* were downregulated, while the expression of *BAG1, BAG3*, and *BAG4 *increased. 

Caspases are a group of proteases produced in an inactive form, which are initiated and regulated by the apoptosis process. In general, caspases involved in apoptosis are classified into two groups, including effectors (-3, -6, and -7) and initiators (caspase-8, -9, and -10) (McComb et al., 2019; Poreba et al., 2019). When caspases are activated, they alter expression of pro-apoptotic and anti-apoptotic proteins, thereby resulting in apoptosis cells. Moreover, caspases exert their effects using extrinsic and intrinsic pathways (Poreba et al., 2019). 

The present study showed that nisin plays its role in apoptosis by increasing mitochondrial pathways. Caspase-9 is the initiator of the mitochondrial or intrinsic pathway of apoptosis, which is activated by numerous cellular stresses (McIlwain et al., 2013; Pfeffer and Singh, 2018). The intrinsic pathway is regulated by the BCL2 (B-cell lymphoma-2) protein family that contains both anti- and pro-apoptotic members, such as BAX and BH3-only proteins (BID), respectively (Zaman et al., 2014; Lopez and Tait, 2015). Our data showed that the expression of pro-apoptotic BID increased, while that of anti-apoptotic BAX decreased, which suggested that the intrinsic pathway was highly activated. The regulation of irregular growth of cancer cells is one of the ways to treat cancer. According to the results from the present study, it seems that the use of nisin as an inducer of apoptosis could play an effective role in restoring this uncontrolled condition into a normal one. 

## Author Contribution Statement

The authors confirm contribution to the paper as follows: study conception and design: Nahid Zainodini, Mohammad Reza Mirzaei; data collection: Nahid Zainodini; analysis and interpretation of results: Nahid Zainodini, Mohammad Reza Hajizadeh; draft manuscript preparation: Nahid Zainodini, Mohammad Reza Mirzaei. All authors reviewed the results and approved the final version of the manuscript.
